# The role of P2X_3_ receptors in bilateral masseter muscle allodynia in rats

**DOI:** 10.3325/cmj.2016.57.530

**Published:** 2016-12

**Authors:** Petra Tariba Knežević, Robert Vukman, Robert Antonić, Zoran Kovač, Ivone Uhač, Sunčana Simonić-Kocijan

**Affiliations:** 1 Department of Prosthodontics, University of Rijeka School of Dental Medicine and School of Medicine, Rijeka, Croatia

## Abstract

**Aim:**

To determine the relationship between bilateral allodynia induced by masseter muscle inflammation and P2X_3_ receptor expression changes in trigeminal ganglia (TRG) and the influence of intramasseteric P2X_3_ antagonist administration on bilateral masseter allodynia.

**Methods:**

To induce bilateral allodynia, rats received a unilateral injection of complete Freund’s adjuvant (CFA) into the masseter muscle. Bilateral head withdrawal threshold (HWT) was measured 4 days later. Behavioral measurements were followed by bilateral masseter muscle and TRG dissection. Masseter tissue was evaluated histopathologically and TRG tissue was analyzed for P2X_3_ receptor mRNA expression by using quantitative real-time polymerase chain reaction (PCR) analysis. To assess the P2X_3_ receptor involvement in nocifensive behavior, two doses (6 and 60 μg/50 μL) of selective P2X_3_ antagonist A-317491 were administrated into the inflamed masseter muscle 4 days after the CFA injection. Bilateral HWT was measured at 15-, 30-, 60-, and 120-minute time points after A-317491 administration.

**Results:**

HWT was bilaterally reduced after the CFA injection (*P* < 0.001). Intramasseteric inflammation was confirmed ipsilaterally to the CFA injection. Quantitative real-time PCR analysis demonstrated enhanced P2X_3_ expression in TRG ipsilaterally to CFA administration (*P* < 0.01). In comparison with controls, the dose of 6 μg of A-317491 significantly increased bilateral HWT at 15-, 30-, and 60-minute time points after the A-317491 administration (*P* < 0.001), whereas the dose of 60 μg of A-317491 was efficient at all time points ipsilaterally (*P* = 0.004) and at 15-, 30-, and 60-minute time points contralaterally (*P* < 0.001).

**Conclusion:**

Unilateral masseter inflammation can induce bilateral allodynia in rats. The study provided evidence that P2X_3_ receptors can functionally influence masseter muscle allodynia and suggested that P2X_3_ receptors expressed in TRG neurons are involved in masseter inflammatory pain conditions.

Temporomandibular disorders (TMDs) are a group of musculoskeletal conditions affecting the masticatory system and resulting in disability and pain. Common TMD symptoms include reduced mouth opening and jaw mobility, jaw locking, and temporomandibular joint (TMJ) noises. The most prominent symptom is pain typically located in the masseter muscles (1).

The cause of TMDs remains unclear. Recent studies on TMDs focus on changes in pain mechanisms rather than etiological factors, because even patients with negligible anatomical changes may develop pain sensations, and unilateral masseter or TMJ inflammation can provoke bilateral and/or referred hyperalgesia and/or allodynia (2-4).

New findings point out the key role of receptor-regulated ion channels in peripheral and central neurons in pain mechanisms. Trigeminal ganglion (TRG) neurons express different receptor types and their expression changes, and their ability to bind ligands characteristic of tissue damage imply their significance in pain transmission and, therefore, in the etiopathogenesis of TMDs (5-7).

The purinergic signaling system, especially P2 and P2X receptor families, is one of the most important receptor systems involved in pain mechanisms. There are at least 12 P_2_X purinoceptors expressed in the central and peripheral neurons and involved in neurotransmission, neuromodulation, and neuromuscular transmission. They are all ATP-gated cation channels, although there are also other agonists with restricted subtype selectivity (8,9).

Although all P2X receptors are present in sensory neurons, P2X_3_ receptors are expressed at the highest levels, especially in nociceptive C and Aδ fibers (10,11). Recent studies have shown that P2X_3_ plays an important role in different pain states including migraine pain states (12), diabetic neuropathic pain (13), bone cancer pain conditions (14), and many others. P2X_3_ has recently been identified as one of the most important in still unclear signaling mechanisms underlying orofacial pain (15,16). The stretching and eccentric contraction of the masseter muscle induces hyperalgesia and up-regulation of P2X_3_ expression leading to enhanced nociceptor responsiveness to increased adenosine triphosphate (ATP) levels within occurring myofiber damage (17). A recent study determined P2X_1_, P2X_3_, and P2X_2/3_ receptors involvement in carrageenan-induced TMJ inflammatory hyperalgesia (18). Conversely, there are data suggesting that P2X purinoceptors, especially P2X_3_ and P2X_2/3_, could be involved in antinociception (19,20).

Although the underlying mechanism for masseter muscle pain is still unclear, recent studies highlight the involvement of various receptors in the etiopathogenesis of TMDs and propose various antagonist and/or agonists as possible therapeutic agents. Previous studies have investigated receptor-regulated ion channel expression in various pain models, but not in the masseter inflammation model. As masseter pain is the most prominent symptom in patients with TMDs, and P2X_3_ receptors seem to be involved in nociception originating from orofacial region, we decided to investigate the expression of P2X_3_ receptors in TRG after eliciting bilateral allodynia induced by unilateral masseter inflammation and effects of direct unilateral intramuscular administration of two doses of specific P2X_3_ antagonist on bilateral pain response. Out hypothesis was that bilateral masseter allodynia after unilateral masseter inflammation results from P2X_3_ receptor up-regulation at TRG level and that unilateral intramasseteric administration of P2X_3_ antagonist leads to bilateral masseter allodynia attenuation.

## MATERIAL AND METHODS

### Experimental animals

Experiments were performed on 10- to 12-week-old male Wistar rats weighing 250-300 g (n = 52). Before any animal manipulation, the animals were housed under controlled environment for 7 days to avoid possible stress influence due to transport from the breeding facility. The rats were housed in groups of two to three per cage under a controlled ambient at a temperature of 20°C to 24°C, humidity of 45% to 65%, in a 12h/12h light/dark cycle, and with access to food and water *ad libitum*. All efforts were made to minimize animal suffering and reduce the number of animals. All experimental protocols involving animals were performed in accordance with the Croatian regulations (Official Gazette 135/06, 37/13, and 47/09) and approved by the Croatian Ministry of Agriculture, Cabinet for Veterinary and Food Safety, and the School’s Ethics Committee.

The experiments were performed in two phases. In the first phase, bilateral pain model was established by inducing unilateral masseter inflammation in 16 animals. Eight of these 16 animals received complete Freund’s adjuvant (CFA) and served as the experimental group. The other 8 animals received saline solution and served as the control group. Twelve of these 16 animals (6 from the experimental and 6 from the control group) were subsequently subjected to tissue isolation. The masseter muscle was isolated for histopathological examination and TRG was isolated for quantitative real-time polymerase chain reaction (PCR) analysis. In the second phase, the effect of P2X_3_ receptor antagonist administration on the nocifensive behavior was examined in 36 animals (Table 1).

**Table 1:** Number of animals included in the testing of the effect of P2X_3_ receptor antagonist administration*

	P2X_3_ receptor antagonist administration					
	sham rats			CFA-injected rats		
	saline	D6	D60	saline	D6	D60
No. of rats	6	6	6	6	6	6

* Abbreviations: CFA - complete Freund’s adjuvant; D6 - dose of 6 µg/50 µL of P2X_3_ receptor antagonist A-317491; D60 - dose of 60 µg/50 µL of P2X_3_ receptor antagonist A-317491.

### Bilateral pain induced by unilateral masseter muscle inflammation

In the first phase, all animals (n = 16) were submitted to masseter inflammation induction as described in previous studies (3,21,22). Briefly, animals were anesthetized with 4% isoflurane (Forane, Abbott Laboratories Ltd, Queenborough, UK) in O_2_:N_2_ = 1:2 mixture to abolish righting and corneal reflexes. Eight animals were injected with 50 μL CFA (Sigma F5881, Sigma-Aldrich, Saint Louis, MO, USA, 0.5 mg/mL, heat killed Mycobacterium tuberculosis suspended in oil:saline at 1:1 emulsion) in the mid-region of right masseter muscle. The exact injection site was established by palpating the masseter muscle between the angle of the mandible and the zygomatic bone. Upon contacting the mandible, the needle was slowly withdrawn into the mid-region of the masseter muscle. CFA and saline administrations were made via a 27-gauge needle and completed within 5-10 seconds. Animals in the control group (n = 8) received 0.9% saline injection in the same manner. All solutions were freshly prepared before use. Animals were closely monitored on daily basis for evidence of edema after injection of CFA or saline. Development of masseter inflammation was examined by a histopathological method. Behavior assessment was performed as the next step to confirm bilateral allodynia development.

*Behavior assessment.* We defined mechanical allodynia as the presence of a nocifensive behavioral response evoked at a mechanical threshold that previously did not evoke a nocifensive response. Changes in nocifensive behavioral responses were evaluated by using previously described methods (3,23,24). To confirm bilateral allodynia, the nocifensive behavior response after unilateral CFA or saline injection into the masseter muscle was measured bilaterally at different time points using a von Frey anesthesiometer (VFA; type 2391, IITC Inc., Woodland Hills, CA, USA) with the tip size of 1.0 mm. The threshold was defined as the lowest force necessary to evoke an active withdrawal of the head from the probing tip. The probing tip attached to the VFA was applied to the mid-region of the masseter muscle five times at one minute intervals; the average value of these five measurements was considered the withdrawal threshold.

Before the VFA measurements, animals were accustomed to stand unrestrained on the experimenter’s glove. Mechanical thresholds were then tested by probing the masseter muscles bilaterally before and on Day 4 after CFA or saline injection, when the nocifensive response is most prominent (3). A baseline mechanical threshold for evoking the head withdrawal responses was determined 15 minutes before the CFA or saline injection. All behavioral tests were conducted under blind conditions, ie, by an investigator blind to grouping and drug administration.

### Administration of P2X_3_ receptor antagonist

We initially examined the effect of intramasseteric injection of either P2X_3_ receptor antagonist or saline on the baseline head withdrawal threshold (HWT) in 36 animals (Table 1) (3,25). Animals were lightly anesthetized with 2% isoflurane in O_2_:N_2_ = 1:2 mixture before the administration of P2X_3_ receptor antagonist A-317491 (A2979, Sigma-Aldrich, Saint Louis, USA), which is a selective P2X_3_ antagonist with very limited penetration to the central nervous system (CNS) (26). The P2X_3_ receptor antagonist A-317491 was administered at the dose of either 6 µg/50 µL (D6; n = 6) or 60 µg/50 µL (D60, n = 6). The animals in the control group (n = 6) were administered 50 µL of saline solution. The HWT was measured before and 15, 30, 60, and 120 minutes after the A-317491 or saline administration.

To evaluate the effect of P2X_3_ receptor antagonist on bilateral mechanical threshold after unilateral masseter inflammation (Table 1), the animals were unilaterally injected with CFA under 4% isoflurane anesthesia. On Day 4 after the intramasseteric CFA injection, the bilateral nocifensive response was measured to verify the development of bilateral mechanical allodynia. After the behavioral evaluation, each animal was lightly anesthetized with 2% isoflurane. The P2X_3_ receptor antagonist A-317491 was diluted in distilled water and administered in two different doses, ie, 6 µg/50 µL (D6; n = 6) or 60 µg/50 µL (D60; n = 6). The sham rats in the control group (n = 6) were injected with 0.9% saline in the corresponding volume. All solutions were freshly prepared before use, and the dose and volume of P2X_3_ antagonist were based on previous studies (15,27). The A-317491 was administered unilaterally to the mid-region of the right masseter muscle. The exact injection site was established by palpating the masseter muscle between the angle of the mandible and the zygomatic bone. Upon contacting the mandible, the needle was slowly withdrawn into the mid-region of the masseter muscle. CFA, A-317491, and saline were administered via a 27-gauge needle within 5-10 seconds. The HWT was re-evaluated at 15, 30, 60, and 120 minutes after the A-317491 injection (3,23).

**Tissue isolation, preparation, and analysis**

On Day 4 after the unilateral intramasseteric administration of CFA or saline (Table 1), the final behavioral evaluation was performed. The rats were decapitated and tissue isolation was performed in 6 rats from each group. Bilateral masseter muscles were harvested for histopathological inflammation assessment. Bilateral TRGs were isolated for the evaluation of the P2X_3_ receptor mRNA expression. All tissues were frozen in liquid nitrogen and stored at -80°C until processed.

*Histopathological evaluation of masseter muscle tissue.* Both ipsilateral and contralateral masseter muscles were fixed in 4% paraformaldehide, embedded in paraffin, cut at 40 µm, and stained with hematoxylin and eosin.

*Quantitative real-time PCR.* After rat decapitation and bilateral harvesting of TRG, tissue samples sized to 1 cm2 were frozen in 500 µL of RNAlater (Ambion, Austin, TX, USA) at -80°C. The RNA isolation process involved tissue homogenization performed using a MagNA Lyser instrument (Roche Life Sciences, Mannheim, Germany). The RNA isolation protocol was continued using NucleoSpin RNA kit (Macherey-Nagel, Düren, Germany) according to the manufacturer’s instructions. The concentration of total RNA was determined using Qubit 3.0 instrument and Qubit RNA Broad Range reagent (Life technologies, Carlsbad, CA, USA). Transcription was performed using High Capacity cDNA kit (Applied Biosystems, Foster City, CA, USA). In the process, 500 ng of total RNA was used. Random hexamer primers provided with the kit were used for reverse transcription. cDNA libraries were stored at -25°C prior to the quantification process.

The expression levels of P2X_3_-specific mRNA were measured using Applied Biosystems 7500 Fast Real-Time PCR and Taqman Gene Expression Assays (Applied Biosystems) for P2X_3_ (Rn00579301_m1) as the target and beta-actin (Rn00667869_m1) and GAPDH (Rn01775763_g1) as housekeeping genes (27,28). The evaluation of two different housekeeping genes was used to normalize the values of the cDNA libraries used in the real-time quantification and minimize the RNA isolation and transcription errors. The P2X_3_ relative expression levels were determined by the 2^-ΔΔCt^ method with to compare the expression of genes of interest with that of housekeeping genes.

### Statistical analysis

Data are presented as mean ± standard deviation (SD). All measurements were tested with Kolmogorov-Smirnov test for the normality of distribution. To establish the changes in mechanical HWT before and after the CFA or saline injection, a linear mixed model was used. The one-sample *t* test was used to analyze differences in P_2_X_3_ mRNA expression levels between the two groups. For statistical analysis of behavioral measurements between the groups of rats injected with CFA and P_2_X_3_ antagonist or saline afterwards, a mixed ANOVA was used with time points as within-subjects factor and group as between-subject factor. *P* < 0.05 was considered statistically significant. All statistical tests were performed using SPSS 21.0 software (SPSS Inc., Chicago, SAD).

## RESULTS

### Unilateral intramasseteric CFA injection induced ipsilateral masseter muscle inflammation

The unilateral intramasseteric CFA injection resulted in edema and redness in the injection area. No such changes were observed in the masseter muscle contralateral to the injection site in the CFA-injected rats, nor in the ipsilateral and contralateral masseter muscles in saline-injected rats.

**Figure 1:**
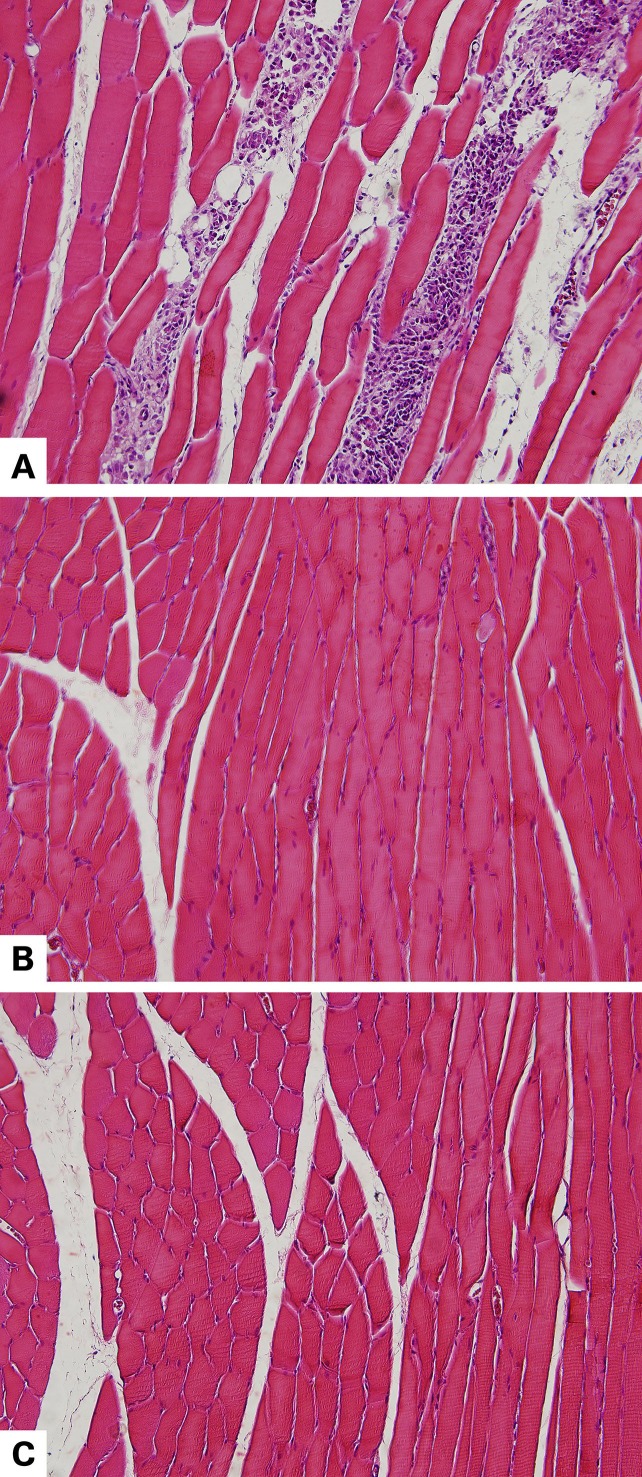
Histopathological examination of the masseter muscle tissue 4 days after unilateral complete Freund’s adjuvant (CFA) injection showed unequivocal inflammation signs (hematoxylin and eosin staining, ×20). The masseter tissue ipsilaterally to CFA injection showed clear signs of inflammation – inflammatory cells infiltration and vacuoles (**A**). Contralaterally to CFA administration, no signs of inflammation are visible in the masseter muscle tissue (**B**). The masseter tissue after saline injection showed no signs of inflammation on either side (**C**).

The intramasseteric CFA injection induced unequivocal histological signs of inflammation including numerous vacuoles and massive infiltration of granular leukocytes at the CFA injection site (Figure 1A). No histological signs of inflammation were perceived in the contralateral masseter muscle of CFA-injected rats nor in the masseter muscles of saline-injected rats (Figure 1B and 1C).

### Bilateral mechanical allodynia induced by unilateral intramasseteric CFA injection

**Figure 2:**
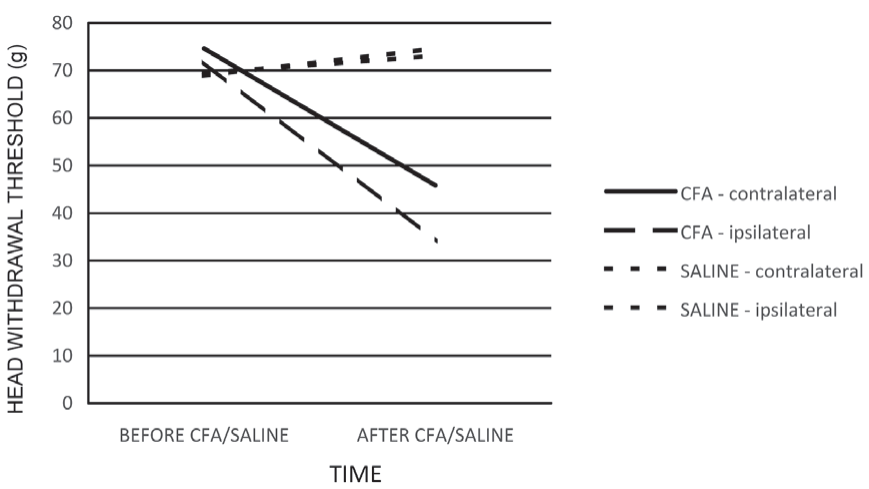
Head withdrawal threshold was statistically lower bilaterally 4 days after complete Freund’s adjuvant (CFA) injection into the masseter muscle region. No differences were observed between the CFA-injected group and saline-injected control group before CFA or saline injection either ipsilaterally or contralaterally. Four days after the CFA injection, a statistically significant differences were observed ipsilaterally and contralaterally (*P* < 0.001 for both) in comparison with the saline-injected control group. The results are presented as mean values, using a linear mixed model. Each group included 8 animals.

The CFA-injected rats (n = 8) showed the reduction in both ipsilateral and contralateral HWT in comparison with saline-injected control rats (n = 8). A statistically significant decrease was identified in bilateral HWT 4 days after the CFA injection (*P* < 0.001; Figure 2). Saline injection in the masseter muscle did not affect HWT on either side (Figure 2).

### Masseter muscle inflammatory allodynia enhanced P2X_3_ expression in the trigeminal ganglion

**Figure 3:**
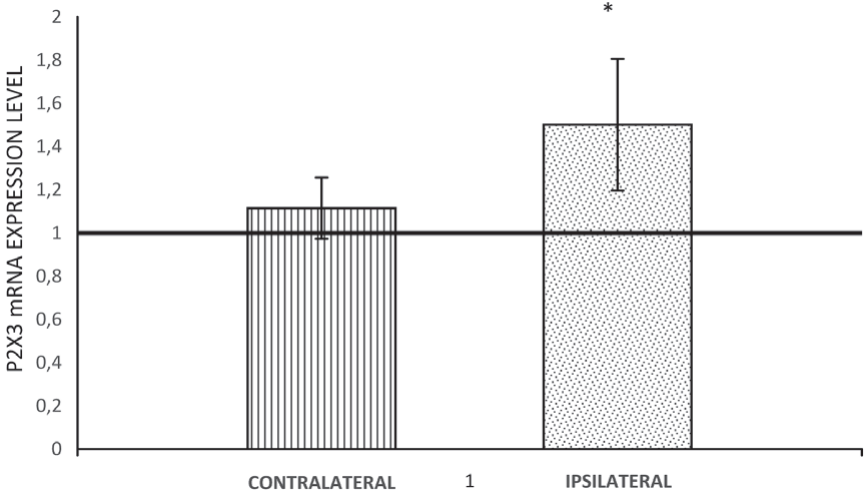
P2X_3_ receptor mRNA expression was up-regulated in ipsilateral trigeminal ganglia (TRG). A statistically significant increase in P2X_3_ receptor mRNA expression was observed at TRG ipsilaterally to the masseter muscle injected with complete Freund’s adjuvant (CFA) in comparison to P2X_3_ expression in ipsilateral TRG in control animals (*P* = 0.010). Although the P2X_3_ receptor mRNA expression was elevated, no statistically significant differences were observed between experimental and control groups at TRG contralaterally to the CFA-injected masseter muscle (*P* = 0.105). Results are presented as median ± standard deviation. Each group included 6 animals. Asterisk indicates a statistical significance of *P* < 0.05 (one-sample *t* test) between the CFA- and saline-injected groups.

Changes in the expression levels of P2X_3_ receptor mRNA in both ipsilateral and contralateral TRGs after the unilateral CFA injection into the masseter muscle were determined by the quantitative real-time PCR technique. Bilateral TRGs were harvested 4 days after the CFA injection. A statistically significant increase in the P2X_3_ mRNA levels was found in the TRG ipsilaterally to CFA injection (*P* = 0.010; Figure 3) in comparison with the controls. Contralateral P2X_3_ mRNA levels were elevated, but they did not show significant differences in comparison with the controls (*P* = 0.105; Figure 3).

### Unilateral administration of P2X_3_ receptor antagonist attenuated bilateral masseter muscle allodynia

The specific P2X_3_ receptor antagonist A-317491 was unilaterally administered into the masseter muscle of rats to test the possible effect of P2X_3_ receptor changes on bilateral allodynia induced by unilateral masseter inflammation.

**Figure 4:**
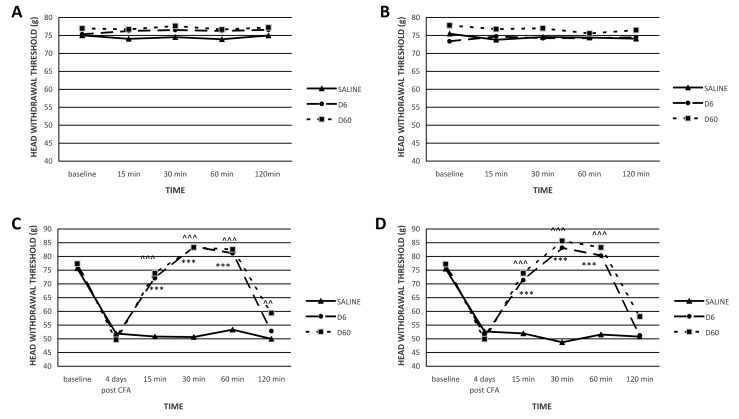
Unilateral administration of A-317491 in two different doses (6 µg [D6] and 60 µg [D60]) attenuated bilateral masseter inflammatory allodynia at 15-, 30-, and 60-minute time points. No significant changes were observed between the groups in head withdrawal threshold (HWT) measurements either ipsilaterally or contralaterally to A-317491 or saline injection in sham rats (**A, B**). Ipsilaterally to complete Freund’s adjuvant (CFA) injection, significant HWT increase in comparison with controls was observed in D6 groups at 15-, 30-, and 60-minute time points after intramasseteric A-317491 injections (*P* < 0.001). At 120-minute time point, the difference between the D6 and saline-injected groups was not significant (*P* = 0.491). In the D60 group, significant differences were found at all observed time points in comparison with controls (*P* ≤ 0.004). Differences between D6 and D60 groups appeared at 120-minute time point (*P* = 0.042) (**C**). In the contralateral masseter muscle, the HWT increase occurred at 15-, 30-, and 60-minute time points in both A-317491 groups compared with controls. No significant difference was observed at 120-minute time point. No significant difference was found between D6 and D60 groups (**D**). Data are presented as mean values (mixed ANOVA). Each group included 6 animals. Asterisks indicate statistical significance of *P < .05; **P < 0.01; ***P < 0.001 for difference between D6 and saline-injected groups after A-317491 administration. Caret indicates a statistical significance of ^*P* < 0.05; ^^*P* < 0.01; ^^^*P* < 0.001 for difference between D60 and saline-injected groups after A-317491 administration.

To exclude the possible influence of the P2X_3_ receptor antagonist A-317491 on baseline HWT in untreated rats, HWT was measured bilaterally before and after the intramasseteric injection of the P2X_3_ receptor antagonist A-317491 in the D6 (n = 6) and D60 (n = 6) groups of rats and before and after the intramasseteric injection of saline in control rats. No statistically significant differences were found between the A-317491-injected and saline-injected groups of rats at any observed time point either ipsilaterally or contralaterally to A-317491 or saline injection (Figures 4A and 4B). Neither dose of A-317491 or saline influenced the bilateral baseline values of HWT at any observed time points.

On Day 4 after the CFA injection, HWT values significantly decreased in all groups (*P* ≤ 0.001 for all), reconfirming the allodynia occurrence both ipsilaterally and contralaterally to the CFA injection (Figures 4C and 4D). Ipsilaterally to CFA and A-317491 or saline injections, a significant increase in HWT occurred 15, 30, and 60 minutes after the A-317491 injection in the D6 group in comparison with the saline-injected control group (*P* < 0.001), whereas in the D60 group, a significant increase in HWT occurred at 15, 30, 60, and 120 minutes after the A-317491 injection in comparison with saline-injected control group (*P* < 0.001 at 15-, 30-, and 60-minute time points; *P* = 0.004 at 120-minute time point). No significant differences were noticed between D6 and D60 groups at any time points except at 120-minute time point (*P* = 0.042; Figure 4C).

Contralaterally to CFA and A-317491 or saline administration, allodynia attenuation occurred at 15-, 30-, and 60-minute time points in both D6 and D60 groups in comparison with the saline-injected control group (*P* < 0.001). No significant differences were noticed at 120-minute time point (*P* = 0.990 for D6 group, *P* = 0.077 for D60 group). Between D6 and D60 groups, no statistically significant differences were found at any time point (Figure 4D).

## DISCUSSION

We aimed to determine the role of P2X_3_ receptors in pathophysiological mechanisms underlying bilateral allodynia induced by unilateral masseter inflammation and found that unilateral masseter inflammation enhances P2X_3_ receptor expression within neurons of the ipsilateral TRG. No changes in the P2X_3_ receptor expression were observed in the contralateral TRG neurons.

P2X_3_ receptors are involved in many pain states within the orofacial area. Hyperalgesia induced by occlusal interference resulted in enhanced P2X_3_ mRNA expression and increased frequency of P2X_3_-immunoreactive small-sized TRG neurons and was attenuated by P2X_3_ antagonist administration (29). Pain transmission in tooth is regulated through ATP transport in odontoblasts and signal transduction to P2X_3_ receptors on pulp axons (30). Noma et al (15) reported increased P2X_3_ expression in masseter hyperalgesia following excessive masseter contraction. As mentioned earlier, eccentric muscle contraction and muscle stretching can lead to hyperalgesia and P2X_3_ up-regulation in afferent TRG neurons (17). Unilateral TMJ arthritis enhanced P2X_3_ receptors expression in the ipsilateral small TRG neurons 15 days after CFA injection. Unilateral P_2_X agonist administration resulted in bilateral TMJ pain (17). It has been suggested that lip inflammation leads to increased P2X_3_ receptors expression in TRG mediated by calcitonin gene-related peptide and could be involved in ectopic mechanical allodynia (31). In addition, some previous studies have determined enhanced P2X_3_ receptors expression in dorsal root ganglion (DRG) after muscle inflammation (32). However, some studies claim that P2X_3_-expressing TRG neurons considerably differ from DRG afferent neurons, suggesting differences in pain transmission depending on the location of pain occurrence, and that the underlying mechanisms of pain originating from different body regions are unique (21,22).

We investigated whether unilateral masseter inflammation led to changes in P2X_3_ receptor expression at bilateral TRG level. Our quantitative real-time PCR analysis results showed increased P2X_3_ receptor expression in TRG both ipsilateral and contralateral to CFA injection, but only the changes in the ipsilateral inflamed TRG showed statistical significance in comparison with controls, leading us to a conclusion that the up-regulation of P2X_3_ receptors is related to inflammation and that allodynia in the contralateral non-inflamed masseter muscle probably arises from the changes in expression of some other receptor-regulated ion channels involved in neuromuscular pain transmission. It seems that the inflammation is responsible for changes in P2X_3_ expression at primary neuronal level.

In addition, we demonstrated that ipsilateral administration of P2X_3_ receptor antagonist, A-317491, into the masseter muscle results in bilateral masseter pain alleviation in previously unilaterally CFA-injected rats.

To demonstrate P2X_3_ receptor involvement, we tested the effect of unilateral administration of P2X_3_ receptor antagonist on bilateral nocifensive response in previously CFA-injected rats. Our results showed that unilateral CFA injection in the central region of masseter muscle followed by ipsilateral injection of A-317491 significantly increased the HWT values both ipsilateral and contralateral to CFA injection site. A-317491 decreased nocifensive response on the side ipsilateral to inflammation and A-317491 injection due to its direct influence on P2X_3_ receptors; however, changes also occurred on the contralateral side. Because A-317491 is an antagonist with limited CNS penetration, these data also suggest that unilateral inflammation results in bilateral allodynia due to P2X_3_ receptor enhancement ipsilateral to inflammation, while contralateral allodynia is more likely related to other receptor-regulated ion channel expression changes induced by the CNS pathways that seem to be crucial in bilateral pain development after unilateral inflammation. Recent studies have demonstrated the important role of trigeminal interpolaris/caudalis (Vi/Vc) transition zone and rostral ventromedial medulla (RVM) in processing trigeminal pain arising from masseter muscle inflammation and in the development of bilateral hyperalgesia/allodynia. It has been suggested that contralateral hyperalgesia in the orofacial region is mediated through descending facilitatory mechanisms of the RVM-Vi/Vc circuitry (33-35). It has also been suggested that inhibition of glia and inflammatory cytokines cascade blockage following inflammation provides pain relief (35). Taking into consideration that A-317491 has a poor ability to penetrate the blood-brain barrier, its bilateral pain alleviation effect may arise from its blockage of P2X_3_ receptors at TRG neurons and interrupted cytokines cascade leading to activation of CNS pathways.

The significant difference between D6 and D60 groups only at 120-minute time point ipsilateral to A-317491 administration lead us to the conclusion that the lower A-317491 dose could be enough to obtain analgesic effect, but further experiments are needed to establish the appropriate dose.

An additional finding of our study was that unilateral masseter inflammation led to bilateral masseter pain. Although pain sensation development and mechanisms involved in various pain models have been extensively investigated (15,29,36-38), only limited number of studies focused on bilateral pain (3,16,36,39). A few research projects reported confusing data on whether unilateral inflammation can provoke bilateral nocifensive behavior (36,40), whereas several studies reported that unilateral inflammation resulted in bilateral allodynia (3,41,42).

We managed to establish a bilateral masseter pain model after unilateral inflammation. We used the CFA-induced model of inflammatory pain commonly used in studies investigating orofacial pain (3,43-45). Our findings confirm that unilateral masseter inflammation can cause bilateral nocifensive behavior on Day 4 after CFA injection. We chose the Day 4 time point according to previous studies reporting that most prominent bilateral allodynia occurs 4 days after the CFA injection (3). Pain develops in both inflamed and contralateral non-inflamed masseter area, suggesting the activation of signaling pathways at both local peripheral level and CNS level.

There were several limitations of our study. The number of experimental animals was low and we did not investigate the changes at the CNS level. Also, we used the 60 µg/50 µL dose of P2X_3_ receptor antagonist A-317491, which had already been proved effective in other pain models, ie, we could have used a different, lower dose of A-317491.

In conclusion, our findings demonstrate that unilateral masseter inflammation can induce bilateral allodynia in rats and enhanced P2X_3_ receptors expression in the TRG ipsilateral to inflammation induction, ie, that bilateral masseter allodynia induced by unilateral masseter inflammation is related to the enhanced P2X_3_ receptor expression at TRG ipsilaterally to inflammation. Our study results showed that unilateral P2X_3_ antagonist administration into the masseter muscle led to bilateral alleviation of masseter allodynia, suggesting that P2X_3_ receptors expressed in TRG neurons can functionally influence masseter allodynia and are involved in pain conditions related to masseter inflammation. Contralateral masseter allodynia is likely to be mediated by other receptor-regulated ion channels.
